# Myoinositol plus α-lactalbumin supplementation, insulin resistance and birth outcomes in women with gestational diabetes mellitus: a randomized, controlled study

**DOI:** 10.1038/s41598-021-88329-x

**Published:** 2021-04-23

**Authors:** Rosario D’Anna, Francesco Corrado, Saverio Loddo, Giuseppe Gullo, Loretta Giunta, Antonino Di Benedetto

**Affiliations:** 1 Department of Human Pathology, University of Messina, Messina, Italy; 2 Department of Clinical and Experimental Medicine, University of Messina, Messina, Italy; 3 University Hospital “AOOR Villa Sofia Cervello”, Palermo, Italy

**Keywords:** Health care, Medical research

## Abstract

To verify whether myo-inositol plus α-lactalbumin may reduce insulin resistance and excessive fetal growth in women with gestational diabetes mellitus. In a 12-month period, 120 women with a diagnosis of gestational diabetes mellitus were consecutively enrolled with an allocation of 1:1 in each group and randomly treated with myo-inositol plus α-lactalbumin plus folic acid (treated group) or folic acid (control group) for 2 months. Primary outcome was the variation of insulin resistance through the study evaluated by HOMA-IR. Secondary outcome was the evaluation, through the study, of fetal growth by ultrasound measurements of abdominal circumference centiles and estimated fat thickness. Some clinical outcomes were also considered. After 2 months, in the treated group, a significant reduction in insulin resistance (HOMA values 3.1 ± 1.4 vs 6.1 ± 3.4, p = 0.0002) and fetal growth was shown (Abdominal circumference centiles 54.9 ± 23.5 vs 67.5 ± 22.6, P = 0.006). Among clinical outcomes, a significant decrease in the rate of women who needed insulin (6.7% vs 20.3%, p = 0.03) and of pre-term birth (0 vs 15.2%, p = 0.007) was evidenced. A combination of myo-inositol and α-lactalbumin may reduce insulin resistance and excessive fetal growth.

**Clinical trial registration**: ClinicalTrials.gov, http://www.clinicaltrials.gov, NCT 03763669, first posted date 04/12/2018; last posted date December 06/12/2018.

## Introduction

Gestational diabetes mellitus (GDM) is defined as any degree of glucose intolerance that occurs for the first time or is first detected during pregnancy, but does not fulfil the criteria of overt diabetes^1^. GDM is usually treated with diet and moderate physical activity, but also with psychosocial support which interacts with diet as well as with physical activity^2^. In about 20% of cases insulin administration is needed to control blood glucose, with the aim to avoid GDM complications, such as macrosomia^3^. Some years ago, our group carried out a study^4^ on the use of diet plus myo-inositol, one of the isomers of inositol, to treat GDM, obtaining a significant reduction in insulin resistance compared with the group treated only with diet. In another recent study, Lubin et al.^5^ demonstrated that the use of myo-inositol may reduce the number of women with GDM not controlled by diet that needed insulin. The supplement myo-inositol has been successfully used for the prevention of GDM and its complications too^6^. Myo-inositol may increase insulin sensitivity by making more phosphatidylinositol available,which seems to be a second messenger of insulin^7^, activating glucose transporter (GLUT-4) and increasing the uptake of glucose. Previously, myo-inositol had been used for the treatment of infertile amenorrhoic women affected by Polycystic Ovary Syndrome (PCOS), obtaining regular cycles and pregnancies^8^. However, Montanino Oliva et al.^9^ evidenced that some women with PCOS treated with myo-inositol were resistant to the supplement, probably for an impaired myo-inositol absorption that may result in a reduced clinical effect. To improve myo-inositol bioavailability, a protein of human milk α-lactalbumin was added, as demonstrated by a recent study^9^. In this study, we treated women with GDM with this new compound (myo-inositol plus α-lactalbumin), with the aim to demonstrate a possible reduction effect on insulin resistance and excessive fetal growth.

## Methods

### Participant recruitment

During a period of 12 months, from October 2018 to October 2019, 120 outpatients from 18 to 44 years old attending the Department of Mother and Child of Messina University, with a single pregnancy and without known or suspected fetal congenital abnormality were enrolled consecutively with a diagnosis of GDM after an Oral Glucose Tolerance Test (OGTT) performed at 24–28 weeks gestation, according to the International Association of the Diabetes and Pregnancy Study Group recommendations^10^. In Italy, screening for GDM is not universal, but it depends on risk factors^11^. The OGTT was performed at 24–28 weeks gestation with a 75-g, 2-h glucose tolerance test, with cut-off values of ≥ 92 mg/dl for time 0; ≥ 180 mg/dl after 1 h and ≥ 153 mg/dl after 2 h; at least one out of the three values over or equal to the cut-off was enough for the diagnosis of GDM. Women considered at risk have at least one of the following conditions: previous GDM, pregravid body mass index (BMI) ≥   25 kg/m^2^, fasting plasma glucose at the first prenatal visit between 100 and 125 mg/dL, age ≥ 35 years, previous macrosomia, a family history of type 2 diabetes (T2D) mellitus and some high-risk ethnic groups.

### Mode of intervention and outcomes

It was an open-label trial and a computer randomization was used with an allocation of 1:1 in each group. We expected to recruit 80 women with GDM every year during the trial registration . Since in the same period the number increased to 120, we decided to enroll all the women even if in the registered trial the number was 80. In the treated group (n. 60), 2 g myo-inositol plus 50 mg α-lactalbumin plus mcg 200 folic acid was given twice a day, whereas in the control group (n. 60) mcg 200 folic acid was given twice a day for 2 months. To all women a diet program was given, but if blood glucose levels were not controlled by diet, insulin was added. The protocol was consistent with the principles of the Declaration of Helsinki, and all participants gave their written informed consent. At the time of OGTT and after 2 months of treatment, insulin resistance was calculated by homeostasis assessment of insulin resistance (HOMA-IR), being the difference in HOMA-IR between groups the first main outcome measure. The secondary outcome measure was the difference between groups of fetal sonographic measures, evaluated by ultrasound at diagnosis of GDM and after 2 months of treatment. But after the first cases, it was evident that only abdominal circumference (AC) measure was important, in accordance with a recent study^12^ which demonstrated that in women with GDM AC measure was the unique biometric parameter that progressively increased until 36 weeks gestation. AC value was measured by the same operator and expressed in centiles in accordance with the WHO fetal growth charts^13^, with the aim to normalize the data independently of gestational age. Furthermore, the subcutaneous abdominal fetal adipose tissue thickness was measured at beginning and at the end of the study, using the technique reported by Larciprete et al.^14^. Furthermore, we considered for the final evaluation also clinical outcomes, which were: (a) the number of women who needed insulin, (when we registered the trial, need of insulin was an exclusion criteria, but subsequently, we considered that this might be an interesting clinical outcome^5^ and women who needed insulin were not excluded) (b) the rate of Caesarean Section (CS) in emergency, (c) the rate of Large for Gestational Age (LGA) fetuses, with a birth weight ≥ 95 centile, in accordance with Italian Charts^15^, (d) the rate of pre-term birth (before 259 days of pregnancy from the last menstruation), (e) the rate of hypertensive disorders (blood pressure ≥ 140/90) and of (f) admission to Neonatal Intensive Care Unit (NICU). Serum insulin was measured with an enzyme-linked immunosorbent essay commercial kit.

### Statement

The experimental protocol was approved by the Ethic Committee of Messina University Hospital, with the number 741/2018.

### Statistics

Statistical analysis was performed with IBM SPSS Statistics for Windows (version 22; IBM Corporation, Armonk, NY). Descriptive results of continuous variables are expressed as mean ± SD or n (%). To compare the two groups, the unpaired t test (parametric distributions) or the Mann–Whitney U test (nonparametric distributions) was used. Categorical variables were compared using the χ^2^ test and bivariate associations were estimated using the Pearson’s correlation coefficient. Maternal age, pre-pregnancy BMI and weight gain were adjusted in the regression model.

All statistical comparisons are two-tailed and they were considered significant at the P < 0.05 level.

## Results

All the 120 women enrolled but one completed the study, she missed the second control and delivered in another hospital. General characteristics of the population studied are reported in Table 1. There were no statistical difference in maternal age, pre-gestational Body Mass Index (BMI) and percentage of nulliparas.

**Table 1:** Characteristics of the study groups.

Characteristics	Cases (n. 60)	Controls (n. 59)	p value
Maternal age (years)	34.3 ± 5.5	33.3 ± 6.4	0.35
Pre-gestational BMI (kg/m^2^)	26.4 ± 4.9	27.2 ± 5.9	0.43
Nulliparas (n. & %)	32 (53.3)	32 (54.2)	0.89

In Table 2, primary and metabolic outcomes are reported. There was no difference for gestational age and increased weight at the time of OGTT. About the primary outcome, after 2 months of treatment, in the women treated with only diet, HOMA-IR values were significant decreased in the treated group and the difference was statistically significant (p = 0.007) compared to the control group, even after adjusting for maternal age, pre-pregnancy BMI and weight gain. There was also a statistical difference between groups regarding the rate of women who needed insulin (6.7 vs 20.3, p = 0.03).

**Table 2:** Primary and metabolic outcomes.

Outcomes	Cases (n. 60)	Controls (n. 59)	p value
Gestational age at OGTT (days)	178 ± 12	178 ± 10	0.90
Weight gain at OGTT (kg)	6.7 ± 4.2	7.4 ± 3.6	0.90
Glucose (mg/dl) at TO	91.6 ± 19.6	92.6 ± 10	0.90
Insulin (µU/ml) at TO	23 ± 19.4	19.2 ± 13.2	0.22
HOMA-IR at T0	5.2 ± 4.1	4.5 ± 3.4	0.03
HOMA-IR at T2	3.1 ± 1.4	6.1 ± 3.6	0.0002
Insulin treatment (n. & %)	4 (6.7)	12 (20.3)	0.03

For the secondary outcome, there were no statistical difference between groups at time 0 for CA centile values, whereas after 2 months a significant difference between groups for the CA centiles values was shown (p = 0.03) (Table 3). Furthermore, a significant difference was shown also for abdominal subcutaneous adipose tissue after 2 months between groups (p = 0.02).

**Table 3:** Secondary outcomes.

Outcomes	Cases (n. 60)	Controls (n. 59)	p value
AC centiles T0	69 ± 24	65 ± 26	0.43
AC centiles T2	55 ± 23	67 ± 23	0.03
SATT T0	4.3 ± 1.4	4.4 ± 1.4	0.89
SATT T2	5.7 ± 1.3	6.5 ± 1.8	0.02

*AC* abdominal circumference, *SATT* subcutaneous adipose tissue thickness.

In addition, HOMA values correlated significantly with CA centiles values, both at the beginning of the study and after supplementation with myo-inositol (r = 0.95, p < 0.005). In relation to clinical outcomes (Table 4), a birth weight ≥ 95 centile was less frequent in the treated group (6.7%) than in the control group (11.9%), but the difference was not significant. Also for hypertensive disorders, more cases occurred in the control group (11.9%) compared to the treated group (3.3%), but the difference was not significant. There was no difference between groups for the rate of CS in emergency and admission to NICU. The only outcome statistically significant was the rate of pre-term birth, with no case in the treated group versus 15.2% in the control group (p = 0.007).

**Table 4:** Clinical outcomes.

Outcomes	Cases (n. 60)	Controls (n. 59)	p value
Gestational age at delivery (days)	273 ± 6	268 ± 12	0.002
Birth weight (g)	3219 ± 447	3105 ± 454	0.17
LGA (n. & %)	4 (6.7)	7 (11.9)	0.30
CS in emergency (n. & %)	6 (10)	9 (15.2)	0.40
Hypertensive disorders (n. & %)	2 (3.3)	7 (11.9)	0.30
Pre-term birth (n. & %)	0	9 (15.2)	0.007
Admission to NICU (n. & %)	0	3 (5.1)	0.30

*LGA* large for gestational age, *CS* caesarean section, *NICU* neonatal intensive care unit.

## Discussion

This study has achieved potential important results. After 2 months, in the group treated with a new compound (myo-inositol plus α-lactalbumin) a statistically significant reduction of insulin resistance, number of women needed insulin, fetal abdominal circumference value and subcutaneous adipose tissue thickness was demonstrated. Furthermore, in the treated group no cases of pre-term birth compared with 15.2% of control group, with a difference statistically significant.

There are very few studies on the effects of myo-inositol in improving GDM condition. In particular, this trial confirms two previous similar studies in which a reduced insulin resistance^4^ and number of women needed insulin^5^ was demonstrated. In particular, in the treated group, the number of women who needed insulin was only one third compared with the control group. This is an important goal because insulin treatment is frequently responsible of discomfort in women with GDM for the multiple daily injections, for the risk of hypoglycemia and excessive maternal weight gain. Furthermore, we demonstrated that myo-inositol plus α-lactalbumin may prevent the excessive fetus abdominal circumference growth and subcutaneous adipose tissue thickness that in GDM cases increases progressively^13,14^. It is note-worthy that even if insulin resistance at time 0 was significantly greater in the treated compared with the control group, this data was reversed after 2 months of treatment. There was no significant difference in birth weight between groups, but mean gestational age at delivery in the control group was significantly lower. This latter result was the consequence of an important clinical outcome such as pre-term birth which was found significantly reduced in the treated group compared to the control group. In the myo-inositol group, there was a decreased also for the other clinical outcomes, but probably the number of women enrolled was not enough to obtain a statistically significant difference. In particular, the rate of hypertensive disorders was more than triple in the control group and almost double the cases of birthweight ≥ 95° centile. The exact mechanism of action of myo-inositol is still unclear, and in their review, Croze ML and Soulage CO^16^ suggested that myo-inositol supplementation may increase insulin sensitivity by making more phosphatidylinositol available, which is well-known acting like a second messenger of insulin^7^. This effect seems to improve glucose uptake from the bloodstream to the cells and with a reduced release of free fatty acid from adipose tissue^8^ may contribute to improve insulin sensitivity Furthermore, it has been demonstrated that myo-inositol is able to reduce blood glucose variability in women with GDM^17^, and also lowering glucose level in women affected by diabetes mellitus type 2^18^. In particular, in this kind of women a myo-inositol depletion was demonstrated^19^. Limitations of the study were the limited number of women enrolled and that it was an open-label and not a double-blind study.

## Conclusion

With this study, we have shown that a combination of myo-inositol and α-lactalbumin may reduce insulin resistance and excessive fetal growth in women with GDM. Furthermore, a reduced rate of insulin treatment and pre-term birth was demonstrated in the treated group.

## References

[1] American Diabetes Association (2020). Classification and diagnosis of diabetes. Standard of medical care in diabetes. Diabetes Care.

[2] Gilbert L, Gross J, Lanzi S, Quansah DY, Puder J, Horsch A (2019). How diet, physical activity and psychosocial well-being interact in women with gestational diabetes mellitus: An integrative review. BMC Pregnancy Childbirth..

[3] Hartling L, Dryeden DM, Guthrie A, Muise M, Vandermeer B, Donovan L (2013). Benefit and harms of treating gestational diabetes mellitus: A systematic review and meta-analysis for the U.S. Preventive Services Task Force and the National Institutes of Health Office of Medical Applications of Research. Ann. Intern. Med..

[4] Corrado F (2011). The effect of myoinositol supplementation on insulin resistance in patients with gestational diabetes. Diabet. Med..

[5] Lubin V, Shojai R, Darmon P, Cosson E (2016). A pilot study of gestational diabetes mellitus not controlled by diet alone: First line medical treatment with myoinositol may limit the need for insulin. Diabetes Metab..

[6] Santamaria A (2018). Clinical and metabolic outcomes in pregnant women at risk for gestational diabetes mellitus supplemented with myo-inositol: A secondary analysis from 3 RCTs. Am. J. Obstet. Gynecol..

[7] Saltiel AR (1990). Second messengers of insulin action. Diabetes Care.

[8] Facchinetti F (2020). Experts' opinion on inositols in treating polycystic ovary syndrome and non-insulin dependent diabetes mellitus: A further help for human reproduction and beyond. Expert Opin. Drug Metab. Toxicol..

[9] Montanino Oliva M, Buonomo G, Calcagno M, Unfer V (2018). Effects of myo-inositol plus alpha-lactalbumin in myo-inositol-resistant PCOS women. J. Ovarian Res..

[10] Metzger BE (2010). International association of diabetes and pregnancy study groups recommendations on the diagnosis and classification of hyperglycaemia in pregnancy. International Association of Diabetes and Pregnancy Study Groups Consensus Panel. Diabetes Care.

[11] “Istituto Superiore di Sanità,” Linee Guida per la gravidanza fisiologica. http://www.snlg-iss.it/cms/files/LG_ Gravidanza.pdf. Retrieved Sept 2011.

[12] Brand JS (2018). Gestational diabetes and ultrasound assessed fetal growth in South Asian and White European women: Findings from a prospective pregnancy cohort. BMC Med..

[13] Kiserud T (2017). The World Health Organization fetal growth charts: A multinational longitudinal study of ultrasound biometric measurements and estimated fetal weight. PLoS Med..

[14] Larciprete G (2003). Fetal subcutaneous tissue thickness (SCTT) in healthy and gestational diabetic pregnancies. Ultrasound Obstet. Gynecol..

[15] Bertino E (2010). Neonatal anthropometric charts: The Italian neonatal study compared with other European studies. J. Pediatr. Gastroenter. Nutr..

[16] Croze ML, Soulage CO (2013). Potential role and therapeutic interest of myo-inositol in metabolic diseases. Biochimie.

[17] Pintaudi B (2018). Effects of myo-inositol on glucose variability in women with gestational diabetes. Eur. Rev. Med. Pharmacol. Sci..

[18] Pintaudi B, Di Vieste G, Bonomo M (2016). The effectiveness of myo-inositol and D-chiro-inositol treatment in type 2 diabetes. Int. J. Endocrinol..

[19] Kennington AS (1990). Low urinary chiro-inositol excretion in non-insulin-dependent diabetes mellitus. N. Engl. J. Med..

